# Identifying the Best Marker Combination in CEA, CA125, CY211, NSE, and SCC for Lung Cancer Screening by Combining ROC Curve and Logistic Regression Analyses: Is It Feasible?

**DOI:** 10.1155/2018/2082840

**Published:** 2018-10-01

**Authors:** Qixian Yang, Ping Zhang, Rongqiang Wu, Kefeng Lu, Hongxing Zhou

**Affiliations:** Department of Clinical Laboratory, The Affiliated Changzhou Second People's Hospital of Nanjing Medical University, Changzhou, Jiangsu, China

## Abstract

The detection of serum biomarkers can aid in the diagnosis of lung cancer. In recent years, an increasing number of lung cancer markers have been identified, and these markers have been reported to have varying diagnostic values. A method to compare the diagnostic value of different combinations of biomarkers needs to be established to identify the best combination. In this study, automatic chemiluminescence analyzers were employed to detect the serum concentrations of carcinoembryonic antigen (CEA), carbohydrate antigen 125 (CA125), cytokeratin 19 fragment (CY211), neuron-specific enolase (NSE), and squamous cell carcinoma antigen (SCC) in 780 healthy subjects, 650 patients with pneumonia, and 633 patients with lung cancer. Receiver operating characteristic (ROC) curve and logistic regression analyses were also used to evaluate the diagnostic value of single and multiple markers of lung cancer. The sensitivities of the five markers alone were lower than 65% for lung cancer screening in healthy subjects and pneumonia patients. SCC was of little value in screening lung cancer. After combining two or more markers, the areas under the curves (AUCs) did not increase with the increase in the number of markers. For healthy subjects, the best marker for lung cancer screening was the combination CEA + CA125, and the positive cutoff range was 0.577 CEA + 0.035 CA125 > 2.084. Additionally, for patients with pneumonia, the best screening markers displayed differences in terms of sex but not age. The best screening marker for male patients with pneumonia was the combination CEA + CY211 with a positive cutoff range of 0.008 CEA + 0.068 CY211 > 0.237, while that for female patients with pneumonia was CEA > 2.73 ng/mL, which could be regarded as positive. These results showed that a two-marker combination is more suitable than a multimarker combination for the serological screening of tumors. Combined ROC curve and logistic regression analyses are effective for identifying the best markers for lung cancer screening.

## 1. Introduction

Lung cancer is a malignancy with the highest incidence worldwide. Because the early symptoms of lung cancer are not obvious, most of the patients are diagnosed in intermediate and late stages and have poor prognoses. Screening of lung cancer is an effective way to reduce mortality in high-risk individuals [1–4]. Low-dose spiral computed tomography (CT) examination is the recommended method for screening lung cancer [5]. However, due to the potential radiological hazards, some patients are uncomfortable with CT examination, which limits the application of this technique in routine screening [6]. The detection of serum tumor markers is an important method for the diagnosis of tumors [7]. Some lung cancer-related markers, including carcinoembryonic antigen (CEA), carbohydrate antigen 125 (CA125), cytokeratin 19 fragment (CY211), neuron-specific enolase (NSE), and squamous cell carcinoma antigen (SCC), have been widely reported.

CEA is a broad-spectrum tumor marker that is useful for predicting recurrence and survival rates in many carcinomas, such as colon or gastric cancer [8, 9]. Moreover, when combined with carbohydrate antigen 19-9 (CA19-9), the level of CEA is closely correlated with the survival of patients with pancreatic cancer [10]. CA125 is considered a potential marker for ovarian cancer [11], and the combined detection of CA125 and human epididymis protein 4 (HE4) is effective for screening ovarian cancer [12]. CY211 is mainly expressed in epithelial-derived cells and can be used for staging and prognostic analysis of nasopharyngeal carcinoma [13]. NSE is a highly specific marker for neuronal and peripheral neuroendocrine cells and is elevated in the serum of patients with neuroblastoma, melanoma, and seminoma [14]. SCC is a specific marker for squamous cell carcinoma and is an independent prognostic factor for cervical squamous cell carcinoma [15]. These markers are also closely related to lung cancer, and most of the studies focused on the predictive value of these markers for prognosis and survival rate in lung cancer. These results suggest that these markers are important for prognosis and survival assessment [16–19]. However, few studies have focused on the diagnostic value of the above markers in lung cancer. The low sensitivity of detection of single markers is an important reason [20, 21]. The combined detection of multiple markers is the most common method to increase sensitivity [22].

Nevertheless, the combined detection of too many markers also reduces specificity, leading to a significant increase in the false positive rate. We have observed 30 people who were positive for one of the above-mentioned markers in medical examinations in our hospital in 2015. Only one of them was finally diagnosed with lung cancer by imaging and pathological diagnosis, and tumors were not detected in the other 29 patients. However, 25 out of 29 people showed varying degrees of anxiety, and 20 wanted to retest tumor markers. This not only increases the patients' financial burden but also wastes medical resources. Therefore, although the combined detection of tumor markers is believed to be an effective method to assist in the diagnosis of tumors, we do not think that more markers can lead to higher diagnostic efficiency. How many biomarkers need to be combined to meet the screening requirements? Can the optimal combination be identified among various lung cancer markers? No research has been conducted to address these points. Therefore, in this study, we will use the combination of receiver operating characteristic (ROC) curves and logistic regression analyses [23, 24] to select the best marker combination among the markers mentioned above for screening lung cancer.

## 2. Materials and Methods

### 2.1. Subjects

A total of 633 patients, including 410 males and 223 females, aged 64.2 ± 9.9 years, diagnosed with primary lung cancer and hospitalized at the Affiliated Changzhou Second People's Hospital of Nanjing Medical University from 2016 to 2017 were randomly selected. Because the purpose of this study was lung cancer screening in a common population, except for studying their pathophysiological characteristics, no further histopathological classification was conducted on the lung cancer patients. A total of 650 patients with pneumonia, including 329 males and 321 females, aged 66.2 ± 14.9 years, were also selected within the same period. This group of patients comprised 492 patients with bacterial pneumonia and 158 patients with interstitial pneumonia. Furthermore, 780 healthy subjects, including 550 males and 230 females, aged 62.2 ± 12.6 years, were recruited for physical examination. These subjects had unremarkable blood test, liver/kidney function, and chest X-ray examination. All patients and healthy subjects provided informed consent, and the study was approved by the Ethical Committee.

### 2.2. Methods

#### 2.2.1. Detection of Tumor Markers

Fasting peripheral venous blood samples (3.0 mL) were collected from all patients before treatment and healthy subjects. Serum was separated by centrifugation at 4000 rpm for 10 minutes within 2 hours. CEA, CY211, NSE, and CA125 were detected by an automatic electrochemical luminescence analyzer (Cobas e602, Roche, Germany). The concentration of SCC in serum was measured by an Architect i2000 chemiluminescence analyzer (Abbott, USA). All tests were conducted according to instrument operating manuals.

#### 2.2.2. Comparison with Other Methods

Some articles have also examined the diagnostic significance of the above markers in lung cancer without ROC curves [25–27]. The cutoff values for each marker in those studies were obtained from assay kits provided by manufacturers. However, in our study, we set the maximum point of the Youden index on the ROC curve as the cutoff value. To compare our method with that of other studies, we used the relevant assay kits to determine the cutoff values for each marker as follows: CEA < 3.4 ng/mL, CA125 < 35 U/mL, CY211 < 3.3 ng/mL, and NSE < 15.2 ng/mL. The diagnostic value of marker combinations obtained with or without ROC curves for lung cancer was then compared.

#### 2.2.3. Statistical Methods

SPSS 19.0 software was used in all statistical analyses. All data were subjected to a normality test using the Kolmogorov-Smirnov (K-S) test. Normally distributed data were expressed as $\bar{x}\pm s$. Otherwise, the data were represented as *M* (*P*_25_, *P*_75_). The Mann-Whitney *U* test was used to compare the differences in the expression of tumor markers among groups. Binary logistic regression was applied to calculate the predictive probability of combined biomarkers for the diagnosis of lung cancer. ROC curves were constructed using the predictive probability as a covariate. Areas under the curves (AUCs) were used to evaluate the diagnostic value of each marker combination. An AUC greater than 0.9 indicated excellent diagnostic efficacy. An AUC between 0.7 and 0.9 indicated good diagnostic efficacy. An AUC between 0.5 and 0.7 indicated poor diagnostic efficacy. Finally, an AUC of no more than 0.5 indicated the lack of a diagnostic value of the marker. Using the method suggested by DeLong et al. [28], MedCalc software was used to compare differences in AUCs. Chi-square tests were used to compare percentages, and *P* < 0.05 was considered significantly different.

## 3. Results

### 3.1. The Significance of Single Markers for Lung Cancer Screening

**Figure 1:**
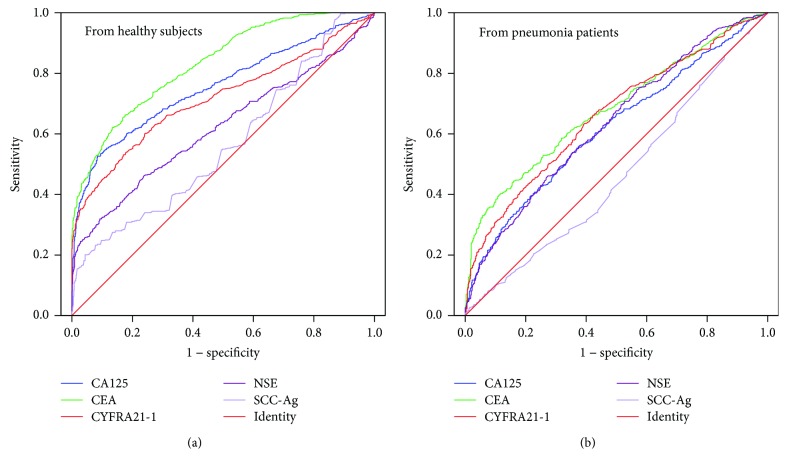
ROC curves of single markers for screening lung cancer in different populations.

Based on the K-S test, the *P* values in all groups were less than 0.05, indicating that the data were not normally distributed. Therefore, the concentration of each marker was represented as *M* (*P*_25_, *P*_75_).  Table 1 shows the concentrations of the five markers in patients with lung cancer and pneumonia and in healthy subjects. The concentrations of all markers in patients with lung cancer and pneumonia were significantly higher than those in healthy subjects. Except for SCC, the other four markers in lung cancer patients were also higher than those in pneumonia patients. The ROC curve and screening value of each marker for identifying lung cancer patients, pneumonia patients, and healthy subjects can be found in Figure 1 and Table 2. When patients with lung cancer were compared with healthy subjects, the value of CEA for lung cancer screening (AUC_CEA_) was significantly higher than that of the other four markers, while NSE and SCC demonstrated little significance for lung cancer screening (AUC < 0.7). For patients with pneumonia, SCC had no value in screening lung cancer, and the significance of the other four markers was also low (AUC < 0.7).

### 3.2. The Value of the Combined Detection of Markers in the Screening of Lung Cancer in Healthy Subjects

**Figure 2:**
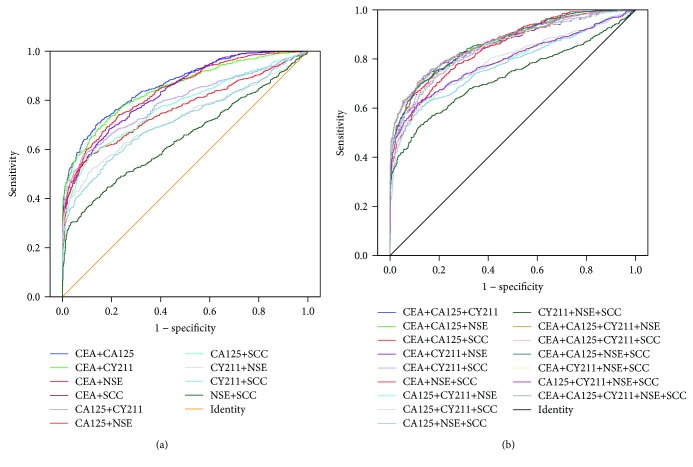
ROC curves of different combinations for lung cancer screening in healthy subjects: (a) combinations with two markers; (b) combinations with multiple markers.

ROC curves for the combined detection of each marker for lung cancer screening in healthy subjects were constructed based on binary logistic regression (Figure 2).  Table 3 shows the value of the combined detection of multiple markers for lung cancer screening. The results displayed that the AUCs for eight combinations (CEA + CA125, CEA + CA125 + CY211, CEA + CA125 + NSE, CEA + CA125 + SCC, CEA + CA125 + CY211 + NSE, CEA + CA125 + NSE + SCC, CEA + CA125 + CY211 + SCC, and CEA + CA125 + CY211 + NSE + SCC) were very close (marked in italics) and were significantly higher than the AUCs for other combinations. There was no significant difference between the minimum AUC (CEA + CA125) (marked by ▲) and the maximum AUC (CEA + CA125 + CY211 + NSE + SCC) (marked by ★) among the eight combinations (*Z* = 1.27, *P* = 0.205). Although CEA + CA125 was less sensitive than CEA + CA125 + CY211 + NSE + SCC (*χ*^2^ = 18.60, *P* ≤ 0.001), its specificity and positive predictive value (PPV) were significantly higher than those of the latter (*χ*^2^ = 65.56 and 20.71, *P* ≤ 0.001), with no significant difference between the negative predictive values (NPV) (*χ*^2^ = 2.50, *P* = 0.114). Moreover, compared with CEA + CA125 + CY211 + NSE + SCC, CEA + CA125 had a high positive likelihood ratio (+LR) and Youden index.

### 3.3. The Value of the Combined Detection of Markers in the Screening of Lung Cancer in Patients with Pneumonia

**Figure 3:**
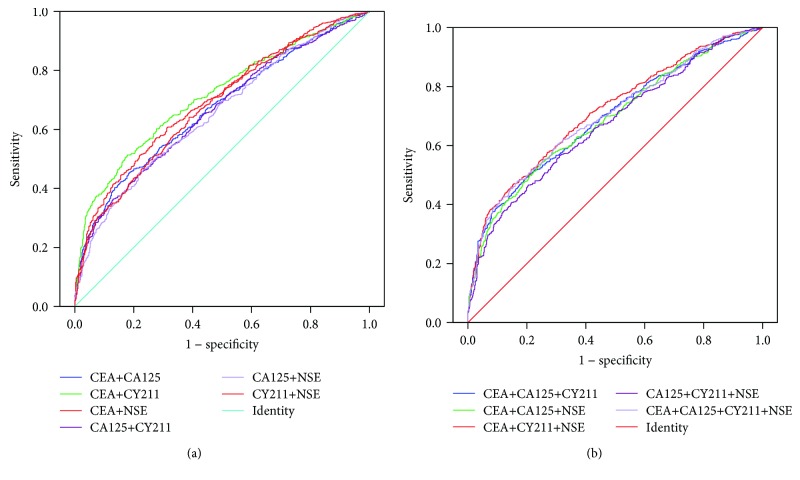
ROC curves of marker combinations for lung cancer screening in patients with pneumonia: (a) combinations with two markers; (b) combinations with multiple markers.

Figure 3 shows the ROC curves of combined markers for lung cancer screening in patients with pneumonia. The value of the combined detection of multiple markers in lung cancer screening is indicated in Table 4. Because SCC was unhelpful in identifying pneumonia and lung cancer (AUC < 0.5), we did not include this marker in this analysis. The results showed that the AUCs of three combinations were greater than 0.7 (CEA + CY211, CEA + CY211 + NSE, and CEA + CA125 + CY211 + NSE) (marked in italics), and there was no significant difference between the AUCs of CEA + CY211 (marked by ▲) and CEA + CY211 + NSE (marked by ★) (*Z* = 0.15, *P* = 0.881). Although the former was less sensitive than the latter (*χ*^2^ = 11.97, *P* ≤ 0.001), its specificity was higher than that of the latter (*χ*^2^ = 20.23, *P* ≤ 0.001), with no significant difference in PPV or NPV (*χ*^2^ = 2.13 and 0.57, *P* = 0.145 and 0.451). The +LR and Youden index of the former were also slightly higher than those of the latter.

### 3.4. The Best Marker Combinations for Lung Cancer Screening Based on Sex and Age

**Figure 4:**
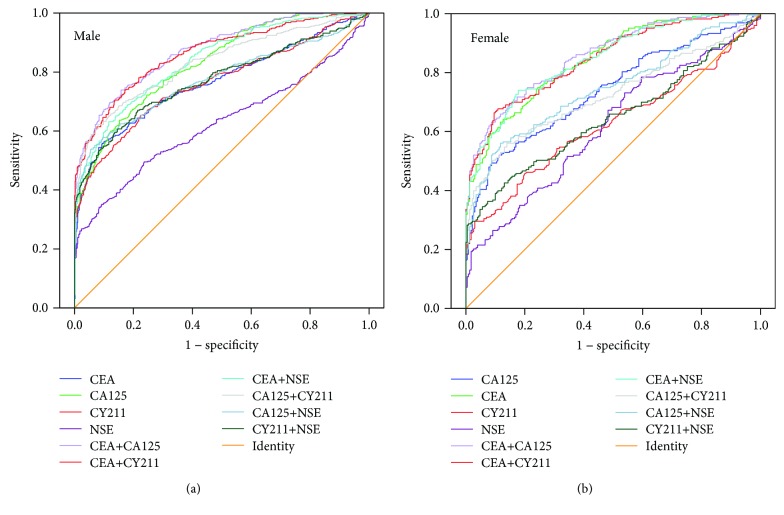
ROC curves of marker combinations for lung cancer screening in healthy subjects grouped by different sexes.

**Figure 5:**
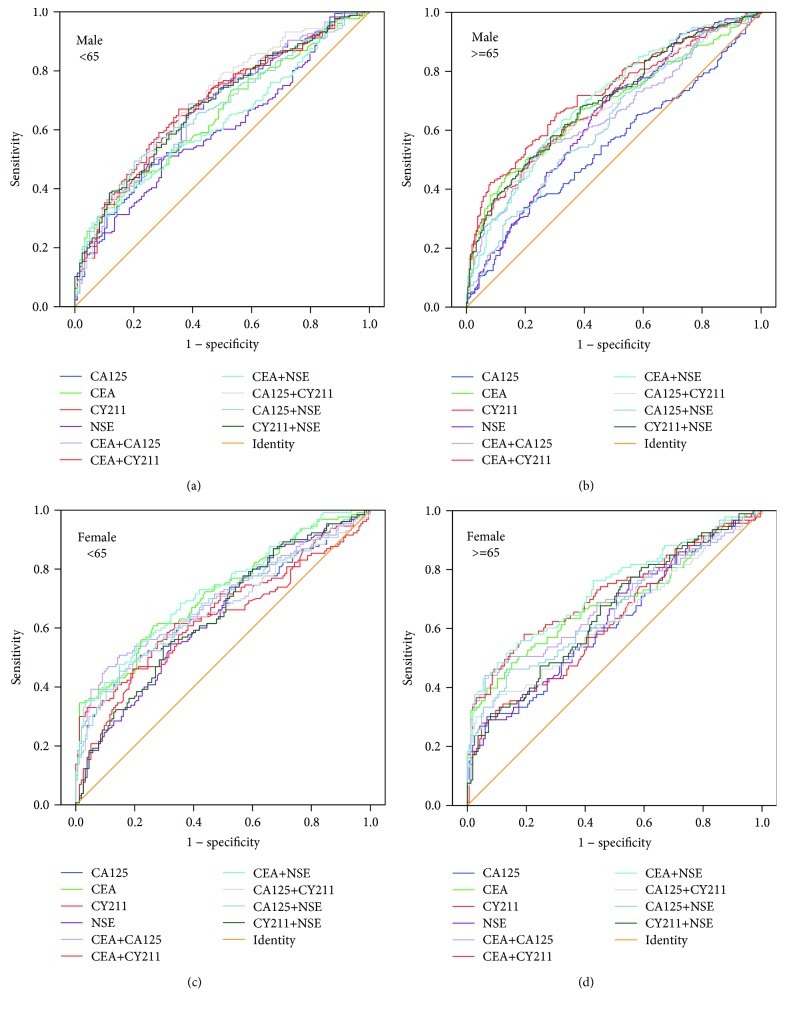
ROC curves of marker combinations for lung cancer screening in patients with pneumonia grouped by different sexes and ages.

The World Health Organization (WHO) defines individuals older than 65 as the elderly (http://www.who.int/healthinfo/survey/ageingdefnolder/en/). We also divided all patients and healthy subjects into two groups based on age, with 65 years as the cutoff value. Each group was further subdivided into two groups according to sex. Finally, patients with lung cancer, patients with pneumonia, and healthy subjects were divided into a total of twelve groups. The number of individuals in each group is shown in Table 5. Then, we performed multivariate binary logistic regression to study the relationship between the incidence of lung cancer and markers of patients with different sexes and ages. We found that, for healthy subjects, the multivariate logistic regression formula was logit*P* = −3.255 + 0.542 CEA + 0.029 CA125 + 0.320 CY211 + 0.037 NSE − 0.569 sex (sex = 1 or 2 indicated males or females, respectively). There was no significance for age to lung cancer in healthy subjects (Wald = 0.001, *P* = 0.973). For patients with pneumonia, the formula was logit*P* = −1.278 + 0.014 CEA + 0.004 CA125 + 0.028 CY211 + 0.019 NSE + 0.529 sex + 0.434 age (sex = 1 or 2 indicated males or females, respectively; for people aged less than 65, age = 1, and for those at the age of 65 or more than 65, age = 2). To analyze the value of the markers in the diagnosis of lung cancer, we performed ROC curve analysis for marker combinations in healthy individuals based on sex and in pneumonia patients based on sex and age (Figures 4 and 5). The AUCs for various combinations of tumor markers for diagnosing lung cancer in different populations are shown in Table 6. We selected the top three combinations according to the AUC in each group (a1, a2, and a3~f1, f2, and f3) (marked in bold type) and compared them for significant differences. In healthy subjects, regardless of sex, the combination CEA + CA125 was useful for screening lung cancer with a large AUC. Moreover, CEA + CY211 was useful for lung cancer screening in male patients with pneumonia. For female patients with pneumonia, a large AUC was obtained with the single detection of CEA. The best marker combination demonstrated no difference in terms of age in any of the subjects.

### 3.5. The Cutoff Range for the Best Marker Combinations for Lung Cancer Screening

Table 7 lists the logistic regression analysis parameters for lung cancer screening in healthy subjects and pneumonia patients based on the combinations CEA + CA125 and CEA + CY211 and the single marker CEA. The fitted equations (logit*P*) are shown in Table 8. The cutoff ranges of logit*P* (cutoff_logit*P*_) and their corresponding incidence (*P*_cutoff_) were calculated at the maximum Youden index. The results showed that for healthy subjects, the incidence of lung cancer was 40.6% when CEA × 0.577 + CA125 × 0.035 > 2.084. For male patients with pneumonia, the incidence of lung cancer was 51.1% when CEA × 0.008 + CY211 × 0.068 > 0.281. In female patients with pneumonia, the incidence of lung cancer was 43.6% when CEA × 0.064 > 0.546 or CEA > 8.52 ng/mL. The results showed that the sensitivity of lung cancer detection was relatively low (<0.6) when calculated based on the maximum Youden index for pneumonia patients. Therefore, the cutoff_logit*P*_ for pneumonia patients was adjusted. Each parameter was recalculated, resulting in a sensitivity close to 0.6 and an increased Youden index (Table 8). The adjusted results revealed that the incidence of lung cancer in male pneumonia patients was 50% when CEA × 0.008 + CY211 × 0.068 > 0.237 and the incidence of lung cancer in female pneumonia patients was 34.8% when CEA × 0.064 > 0.175 (that is CEA > 2.73 ng/mL).

### 3.6. Comparison with Previous Studies for Lung Cancer Screening

In addition to the combination of ROC curve and logistic regression analyses, other methods published in previous studies were employed to evaluate the diagnostic value of the best marker combinations for lung cancer. No ROC curves were used in the previous studies, and the cutoff values of each marker were based on the manufacturer's recommendations. We found that for lung cancer screening in healthy subjects, the sensitivity and specificity of the best marker combination identified by our study (CEA + CA125) were 0.667 and 0.877, respectively, without the use of ROC curves. There was no significant difference regarding sensitivity or specificity between the present method (with ROC curves) and the previous techniques (without ROC curves). Moreover, for lung cancer screening in male pneumonia patients, the sensitivity and specificity of the best marker combination (CEA + CY211) were 0.893 and 0.252, respectively. Compared with those of the present method, a significant increase in sensitivity (*χ*^2^ = 84.16, *P* ≤ 0.001) and an obvious decrease in specificity (*χ*^2^ = 123.08, *P* ≤ 0.001) were observed. However, for female patients with pneumonia, the sensitivity (0.475) of CEA for lung cancer screening was significantly lower (*χ*^2^ = 6.09, *P* = 0.014), whereas the specificity (0.807) was higher (*χ*^2^ = 7.69, *P* = 0.006) based on the non-ROC curve method than on the ROC curve method.

## 4. Discussion

With accumulating studies, a growing number of lung cancer-associated markers, including DNA, RNA, protein, and cell surface markers, have been found [29–34]. The value of each marker in the screening of lung cancer is different. Therefore, we need to establish a method of evaluating these markers. CEA, CA125, CY211, NSE, and SCC are all lung cancer-related biomarkers [35]. The detection of these markers has been automated [36] and is suitable for screening lung cancer on a larger scale. To select the best combination for lung cancer screening, we constructed ROC curves and evaluated the diagnostic value by AUCs. It is necessary to unify multiple variables as a single variable to conduct ROC curve analysis. We used the method suggested by Doseeva et al. [23] and unified different markers for use in the determination of predictive probability through logistic regression and then constructed ROC curves according to the probability.

The value of detection of single markers for identifying patients with pneumonia and lung cancer was low (AUC < 0.7). SCC displayed no effective diagnostic value for lung cancer, regardless of whether it was used in healthy subjects or pneumonia patients. The sensitivities of all markers for lung cancer screening in healthy subjects and pneumonia patients were no more than 0.65. These results indicated that the detection of single markers was of little value in the diagnosis of lung cancer.

Therefore, we combined the markers and studied the value of the combinations for lung cancer screening by logistic regression and ROC curve analyses. For healthy subjects, the AUC increased after markers were combined, but the increase in AUC did not mirror the increase in the number of markers. The main reason is that increased sensitivity inevitably leads to a decrease in specificity. We chose eight combinations with AUCs greater than 0.86, among which only the combination CEA + CA125 consisted of two markers and the rest of the combinations consisted of multiple markers. In general, if the value of detecting multiple markers is equivalent to that of fewer markers, combinations with fewer markers may be preferable for patients. After analysis using MedCalc software, we found no significant difference in AUCs between CEA + CA125 and other multimarker combinations. We also found that the specificity and PPV of CEA + CA125 were higher than those of the other combinations. Therefore, we concluded that for lung cancer screening in healthy subjects, a two-marker combination can reflect the diagnostic value of a multimarker combination, and CEA + CA125 is the best combination without the need for other markers in this study.

For patients with pneumonia, the AUC of none of the marker combinations was significantly improved over the AUC of single markers. Only three combinations displayed a certain value in identifying lung cancer (AUC > 0.7), including a two-marker combination (CEA + CY211) and two multimarker combinations. The AUC of CEA + CY211 was not significantly different from that of the other two combinations. Considering that CEA + CY211 is a two-marker combination, the detection of two markers may be sufficient for lung cancer screening in pneumonia patients, and CEA + CY211 is the best combination.

We further applied multivariate binary logistic regression to analyze the independent clinical value of markers and different ages and sexes. Since we had already found that two-marker combinations have an equivalent diagnostic value to multimarker combinations, we only assessed the difference in ROC curves for markers tested individually and in pairs. We found that CEA + CA125 was the best combination for lung cancer screening in healthy subjects, regardless of age and sex. When the value for CEA and CA125 was 0.577 CEA + 0.035 CA125 > 2.083, the incidence of lung cancer was greater than the cutoff probability (0.406), and the result could be considered positive. In this case, patients should be recommended for further imaging or pathological examinations.

For patients with pneumonia, our study showed that the best screening marker varied by sex but not by age. Both male and female patients with pneumonia displayed reduced sensitivity and had correspondingly high specificity. We thought that the low sensitivity can increase the misdiagnosis rate, and thus, we adjusted the cutoff value to increase the sensitivity. We found that when the value of CEA and CY211 in male patients with pneumonia was 0.008 CEA + 0.068 CY211 > 0.237, the predictive probability for lung cancer was greater than the cutoff probability (0.5), and the result could be considered positive. In this case, the patients should be recommended for further imaging or pathological examinations. For female patients with pneumonia, if the concentration of CEA was more than 2.73 ng/mL, the patients should be recommended for further examinations.

To evaluate the consistency of the present method with other techniques, we referred to previous studies in which the diagnostic value of marker combinations was determined without the use of ROC curves. We found that when lung cancer screening was performed in healthy subjects, the sensitivity and specificity calculated by the current method were consistent with those calculated by previous methods. However, for patients with pneumonia, the screening results using previous methods were different from those of the results using the present technique. In our study, by using ROC curves, the cutoff values for marker combinations could be adjusted to obtain a balance between sensitivity and specificity. This is an important advantage of our study. However, in other studies, the cutoff values were fixed based on the assay kits, and the sensitivity or specificity did not always meet screening requirements. However, in our study, using the non-ROC curve method, the specificity of CEA + CY211 was quite low (0.252) for lung cancer screening in male pneumonia patients and the sensitivity of CEA (0.475) did not meet the screening needs.

Nevertheless, we did not perform further histological classification of the patients with lung cancer. Some studies have found that serological markers can predict the histological classification of lung cancer [37, 38]. We believe that the histological classification of lung cancer should be confirmed by pathological biopsy after the suspected cancer has been diagnosed using serology and imaging. A single serological marker is of little value for predicting the histological subtype of lung cancer.

In addition to the advantage of adjusting the cutoff value to balance the sensitivity and specificity mentioned above, another superiority of our method is the ability to screen for tumor marker combinations. In previous studies, the same tumor markers had been reported to be used in the diagnosis of lung cancer, but the diagnostic value (sensitivity, specificity, etc.) of marker combinations were only mentioned in the articles, and comparisons of diagnostic efficacies of each marker combination to select the optimal combination were not performed, thereby leading to confusions among researchers in choosing suitable marker combinations for screening lung cancer. Our study resolved this problem. In this study, we identified the best combinations using five serological markers for lung cancer screening in different populations and calculated the cutoff ranges of the best combinations by combining ROC curve and logistic regression analyses. Our work suggests that in serological tumor marker screening, a two-marker combination is better than multimarker combinations, and combining ROC curve and logistic regression analyses is feasible for identifying tumor markers. Furthermore, we think that our identified method may have good prospects in other tumors or other nonneoplastic diseases apart from lung cancer.

## Figures and Tables

**Table 1:** Concentrations of serum markers in patients with lung cancer and pneumonia and healthy subjects.

	CEA (ng/mL)	CY211 (ng/mL)	NSE (ng/mL)	CA125 (U/mL)	SCC (ng/mL)
Lung cancer	3.44 (1.88, 12.20)^∗^^#^	3.20 (2.08, 6.24)^∗^^#^	13.76 (11.38, 18.33)^∗^^#^	24.55 (12.09, 63.89)^∗^^#^	0.70 (0.44, 1.24)^∗^
Pneumonia	2.18 (1.39, 3.35)^&^	2.21 (1.57, 3.32)^&^	12.08 (9.78, 14.64)^&^	15.13 (10.16, 31.16)^&^	0.80 (0.50, 1.40)^&^
Healthy	1.27 (0.57, 2.06)	2.10 (1.61, 2.70)	12.37 (10.86, 14.27)	11.60 (8.44, 15.88)	0.68 (0.40, 0.99)

^∗^Compared with healthy subjects, *Z* = 21.47, 13.97, 7.74, 16.56, and 4.22; *P* < 0.01. ^&^*Z* = 14.03, 2.94, 2.23, 10.18, and 7.05; *P* < 0.05. ^#^Compared with pneumonia patients, *Z* = 11.22, 10.03, 8.32, and 7.29; *P* < 0.01.

**Table 2:** Value of single-marker detection for screening lung cancer in patients with pneumonia and healthy subjects.

	SEN	SPE	PPV	NPV	+LR	−LR	Yden	Cutoff	AUC
Pneumonia patients									
CA125	0.539	0.646	0.597	0.590	1.523	0.714	0.185	21.78	0.618
CEA	0.403	0.883	0.770	0.603	3.444	0.676	0.286	4.81	0.681
CY211	0.633	0.606	0.610	0.629	1.607	0.606	0.240	2.57	0.662
NSE	0.461	0.726	0.621	0.580	1.682	0.742	0.187	14.36	0.634
SCC	—	—	—	—	—	—	—	—	0.456
Healthy subjects									
CA125	0.526	0.915	0.834	0.704	6.188	0.518	0.441	22.75	0.756
CEA	0.621	0.867	0.791	0.738	4.669	0.437	0.488	2.74	0.832
CY211	0.539	0.822	0.711	0.687	3.028	0.561	0.360	3.00	0.716
NSE	0.319	0.908	0.738	0.622	3.467	0.750	0.227	16.24	0.620
SCC	0.201	0.956	0.788	0.596	4.568	0.836	0.157	1.60	0.565

SEN: sensitivity; SPE: specificity; PPV: positive predictive value; NPV: negative predictive value; +LR: positive likelihood ratio; −LR: negative likelihood ratio; Yden: Youden index.

**Table 3:** Values of various combinations of markers for lung cancer screening in healthy subjects.

	SEN	SPE	PPV	NPV	+LR	−LR	Yden	AUC
CEA + CA125^▲^	0.755	0.791	0.746	0.799	3.614	0.310	0.546	*0.863*
CEA + CY211	0.761	0.718	0.687	0.788	2.700	0.332	0.479	0.848
CEA + NSE	0.701	0.794	0.734	0.766	3.398	0.376	0.495	0.839
CEA + SCC	0.665	0.833	0.764	0.754	3.991	0.402	0.498	0.835
CA125 + CY211	0.698	0.756	0.699	0.755	2.867	0.399	0.455	0.791
CA125 + NSE	0.621	0.833	0.751	0.730	3.725	0.455	0.454	0.762
CA125 + SCC	0.602	0.873	0.794	0.730	4.742	0.456	0.475	0.774
CY211 + NSE	0.618	0.742	0.660	0.705	2.397	0.515	0.360	0.725
CY211 + SCC	0.578	0.787	0.688	0.697	2.717	0.536	0.365	0.718
NSE + SCC	0.422	0.865	0.718	0.648	3.133	0.668	0.287	0.645
CEA + CA125 + CY211	0.823	0.656	0.660	0.821	2.395	0.270	0.479	*0.866*
CEA + CA125 + NSE	0.795	0.727	0.703	0.813	2.910	0.283	0.522	*0.864*
CEA + CA125 + SCC	0.779	0.759	0.724	0.809	3.231	0.291	0.538	*0.864*
CA125 + CY211 + NSE	0.752	0.687	0.661	0.773	2.404	0.361	0.439	0.791
CA125 + CY211 + SCC	0.724	0.723	0.680	0.763	2.613	0.382	0.447	0.793
CA125 + NSE + SCC	0.671	0.792	0.724	0.748	3.233	0.415	0.464	0.775
CEA + CY211 + NSE	0.798	0.655	0.652	0.800	2.313	0.309	0.453	0.850
CEA + CY211 + SCC	0.777	0.690	0.670	0.792	2.505	0.323	0.467	0.848
CEA + NSE + SCC	0.735	0.762	0.714	0.780	3.081	0.349	0.496	0.842
CY211 + NSE + SCC	0.646	0.709	0.643	0.712	2.220	0.499	0.355	0.727
CEA + CA125 + CY211 + NSE	0.852	0.603	0.635	0.833	2.142	0.246	0.454	*0.867*
CA125 + CY211 + NSE + SCC	0.766	0.655	0.643	0.775	2.222	0.357	0.421	0.794
CEA + CA125 + NSE + SCC	0.814	0.696	0.685	0.821	2.678	0.268	0.510	*0.866*
CEA + CA125 + CY211 + SCC	0.833	0.629	0.646	0.822	2.247	0.266	0.462	*0.867*
CEA + CY211 + NSE + SCC	0.810	0.628	0.639	0.803	2.180	0.302	0.439	0.851
CEA + CA125 + CY211 + NSE + SCC^★^	0.858	0.577	0.622	0.833	2.028	0.246	0.435	*0.868*

**Table 4:** Values of various marker combinations for lung cancer screening in patients with pneumonia.

	SEN	SPE	PPV	NPV	+LR	−LR	Yden	AUC
CEA + CA125	0.656	0.592	0.610	0.638	1.608	0.581	0.248	0.669
CEA + CY211^▲^	0.712	0.585	0.626	0.676	1.715	0.492	0.297	*0.713*
CEA + NSE	0.632	0.657	0.642	0.647	1.842	0.560	0.289	0.698
CA125 + CY211	0.765	0.434	0.568	0.654	1.351	0.543	0.198	0.660
CA125 + NSE	0.692	0.478	0.564	0.615	1.327	0.644	0.170	0.654
CY211 + NSE	0.744	0.477	0.581	0.657	1.422	0.537	0.221	0.676
CEA + CA125 + CY211	0.796	0.417	0.571	0.678	1.366	0.489	0.213	0.693
CEA + CA125 + NSE	0.747	0.443	0.566	0.643	1.342	0.570	0.190	0.689
CA125 + CY211 + NSE	0.829	0.332	0.547	0.667	1.242	0.513	0.162	0.673
CEA + CY211 + NSE^★^	0.796	0.460	0.589	0.699	1.474	0.443	0.256	*0.715*
CEA + CA125 + CY211 + NSE	0.852	0.325	0.551	0.692	1.261	0.457	0.176	*0.701*

**Table 5:** Number of individuals grouped according to sex and age.

		Lung cancer	Pneumonia	Healthy
Male	<65	176	119	284
	≥65	234	210	266
Female	<65	130	160	135
	≥65	93	161	95
Total		633	650	780

**Table 6:** AUCs of various combinations of markers for screening lung cancer in different populations with different sexes and ages.

AUC	Healthy subjects		Pneumonia patients			
	Male	Female	Male	Female		
			<65	≥65	<65	≥65
CEA	0.829	**0.846** ^b3^	0.646	0.681	**0.718** ^e2^	**0.681** ^f3^
CA125	0.766	0.738	0.666	0.547	0.665	0.615
CY211	0.763	0.625	**0.680** ^c3^	0.689	0.637	0.620
NSE	0.624	0.617	0.598	0.640	0.639	0.635
CEA + CA125	**0.873** ^a1^	**0.858** ^b1^	0.674	0.652	**0.700** ^e3^	0.680
CEA + CY211	**0.863** ^a2^	0.846	**0.692** ^c2^	**0.727** ^d1^	0.651	**0.713** ^f2^
CEA + NSE	**0.839** ^a3^	**0.847** ^b2^	0.623	**0.700** ^d2^	**0.728** ^e1^	**0.731** ^f1^
CA125 + CY211	0.832	0.725	**0.698** ^c1^	0.681	0.665	0.627
CA125 + NSE	0.773	0.745	0.671	0.624	0.684	0.664
CY211 + NSE	0.772	0.645	0.680	**0.697** ^d3^	0.645	0.652

a1 vs. a2: *Z* = 1.09, *P* = 0.278; a2 vs. a3: *Z* = 2.67, *P* = 0.008; a1 vs. a3: *Z* = 4.22, *P* < 0.001; b1 vs. b2: *Z* = 1.13, *P* = 0.258; b2 vs. b3: *Z* = 0.25, *P* = 0.806; b1 vs. b3: *Z* = 1.58, *P* = 0.114; c1 vs. c2: *Z* = 0.27, *P* = 0.790; c1 vs. c3: *Z* = 0.84, *P* = 0.404; c2 vs. c3: *Z* = 2.66, *P* = 0.008; d1 vs. d2: *Z* = 1.11, *P* = 0.267; d1 vs. d3: *Z* = 2.07, *P* = 0.039; d2 vs. d3: *Z* = 0.16, *P* = 0.871; e1 vs. e2: *Z* = 0.61, *P* = 0.542; e1 vs. e3: *Z* = 0.95, *P* = 0.342; e2 vs. e3: *Z* = 0.63, *P* = 0.527; f1 vs. f2: *Z* = 0.65, *P* = 0.514; f1 vs. f3: *Z* = 1.66, *P* = 0.097; f2 vs. f3: *Z* = 1.83, *P* = 0.067.

**Table 7:** Parameters of logistic regression for screening lung cancer with the best marker combinations.

		*B*	SE	Wald	*P*	OR
Healthy people	CEA	0.577	0.049	139.37	≤0.001	1.781
	CA125	0.035	0.005	60.16	≤0.001	1.036
	Intercept	−2.465	0.145	287.19	≤0.001	0.085
Male patients with pneumonia	CEA	0.008	0.003	8.20	0.004	1.008
	CY211	0.068	0.017	16.12	≤0.001	1.070
	Intercept	−0.237	0.103	5.25	0.022	0.789
Female patients with pneumonia	CEA	0.064	0.013	23.89	≤0.001	1.066
	Intercept	−0.803	0.109	54.21	≤0.001	0.448

SE: standard error; OR: odds ratio.

**Table 8:** Significance of the best marker combinations for lung cancer screening by ROC curve and logistic regression analyses.

		AUC	Cutoff_logit*P*_	*P*_cutoff_	Logit*P*	SEN	SPE	PPV	NPV	+LR	−LR	Yden
Healthy subjects	CEA + CA125	0.863	−0.381	0.406	−2.465 + 0.577 CEA + 0.035 CA125	0.708	0.855	0.799	0.783	4.883	0.342	0.563
Pneumonia patients (male)	CEA + CY211	0.703	0.044	0.511	−0.237 + 0.008 CEA + 0.068 CY211	0.522	0.784	0.751	0.568	2.417	0.610	0.306
			0^∗^	0.500^∗^		0.617^∗^	0.684^∗^	0.709^∗^	0.589^∗^	1.953^∗^	0.560^∗^	0.301^∗^
Pneumonia patients (female)	CEA	0.692	−0.257	0.436	−0.803 + 0.064 CEA	0.350	0.969	0.887	0.682	11.290	0.671	0.319
			−0.628^∗^	0.348^∗^		0.592^∗^	0.713^∗^	0.589^∗^	0.716^∗^	2.063^∗^	0.572^∗^	0.305^∗^

^∗^Recalculated parameters after adjusting cutoff_logit*P*_.

## Data Availability

The data used to support the findings of this study are available from the corresponding author upon request.
